# Speaking of sepsis: semantics, syntax, and slang

**DOI:** 10.3389/fmed.2023.1250499

**Published:** 2023-10-24

**Authors:** Tim J. J. Inglis

**Affiliations:** ^1^Western Australian Country Health Service, Perth, WA, Australia; ^2^Schools of Medicine and Biomedical Sciences, University of Western Australia, Crawley, WA, Australia; ^3^Departments of Microbiology, PathWest Laboratory Medicine WA, QEII Medical Centre, and Fiona Stanley Hospital, Nedlands, WA, Australia

**Keywords:** sepsis, bacteraemia, bacterial bloodstream infection, diagnosis, laboratory medicine, machine learning and AI, immunophenotyping

## Abstract

Medical language is in a constant state of evolution. Its grammar and vocabulary are not fixed by rigid rules. The interdisciplinary field of sepsis has become a meeting point for new insights arising from advances in systems biology, epidemiology, mechanistic understandings of disease process and antimicrobial interventions. This convergence has gained from our recent experience of SARS-CoV-2 infection and COVID-19 and possibilities inferred from emerging information technology. Biomedical descriptors have diverged along disciplinary lines creating an unfortunate disconnect between clinical and laboratory-based terminology. The resulting confusion between clinically determined sepsis and laboratory verified bloodstream infection raises practical questions that affect daily operational processes in the ward, clinic and laboratory. There is an urgent need to understand how the clinical sepsis pathway and corresponding clinical laboratory workflow can be better aligned as a single coherent entity. There is also an implicit need to understand how this process should produce actionable information in a timely and orderly manner, and identify residual obselete terminology that has crept into common usage. A widely accepted sepsis epistemology, ontology and heuristic will help us improve our clinical management of sepsis.

## Introduction

One of the many consequences of the COVID-19 pandemic was to put developments in sepsis management on hold while global health turned its hand to the immediate existential threat. Now that SARS-CoV-2 is no longer recognised as an international health emergency, we can pick up where we left sepsis in pre-COVID times. Pandemic-generated advances in biotechnology, health informatics, therapeutics and vaccines serve to highlight how far sepsis has fallen behind. Meanwhile, there has been no substantive progress on clinical definition, no new single laboratory biomarker, despite the rising tide of antimicrobial resistance among the commoner causal bacteria. Our local attempts at a practical definition of sepsis have stalled at the intent stage: is it for clinical recognition, or epidemiological surveillance? Indeed, the most recent local gap analysis omitted laboratory data and data linkage processes in its plans to implement a new clinical care standard.

### The bad language of sepsis

**Figure 1:**
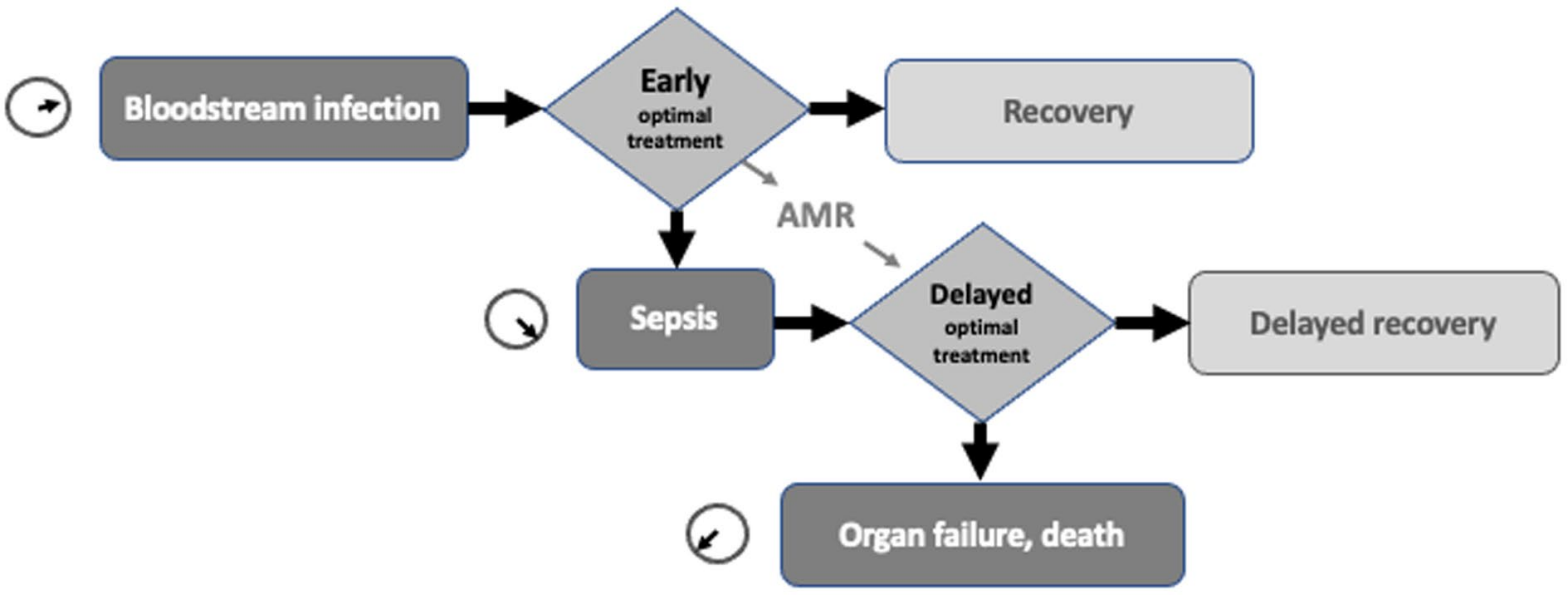
Schematic diagram showing the proposed interrelationship of bloodstream infection, sepsis, their time-critical nature and the delay to optimal therapy caused by antimicrobial resistance (AMR).

It seems that one of the key problems with sepsis is that different professional groups use the same word to describe distinct processes without realising the mismatch. A further source of confusion is the difference in terms used for this complex, dynamic process. At the visible, front end of the patient’s sepsis journey is a collection of clinical signs and symptoms that describe a patient on the slippery slope towards organ failure and death. In the rear stands laboratory medicine with its set of descriptors for bloodstream infection (BSI). It is unclear whether this is one idea described by two languages, or two ideas converging on verbally ambiguous territory? While sepsis and BSI overlap, they are not one and the same thing. Bloodstream infection can be verified by conventional culture methods, but may not progress to established sepsis (See Figure 1). Sepsis, for a variety of reasons, may occur without a positive blood culture. Yet at a recent major meeting speakers switched between sepsis and bloodstream infection as if the two were interchangeable. Both terms appeared in the session title (1). The language has been confused even further by a digression into the systemic inflammatory response syndrome (SIRS) (2), which fell out of favour with introduction of the Sepsis-3 consensus guideline (3). Some colleagues find it hard to give up use of Severe Sepsis and even slip in informal terms to describe borderline patients, such as ‘a bit SIRSy’. These terms may not fit the grammar rules of sepsis, but they underline the inadequacy of current usage and, like all slang, meet an unfilled linguistic need.

### Semantic alignment

There is an urgent need to understand how the clinical sepsis pathway and its corresponding clinical laboratory workflow can better align as a single coherent entity (4). Sepsis is a time-critical medical emergency. At the heart of sepsis management is a trade-off between effective treatment and accurate diagnosis. Whether or not we agree with the proposition that each hour’s delay to effective treatment carries an increased risk of fatality, the emphasis is on early, effective intervention (5). While current clinical definitions of sepsis are at their most reliable after the event, microbiology laboratory results have little bearing on initial clinical decisions. Neither the current concept of sepsis nor laboratory confirmation of bloodstream infection offer much help to the physician who has to make a call on presumptive antimicrobial therapy during the early stages of bloodstream infection. In teaching hospitals, where subject matter expertise reaches critical mass, alignment between the patient journey and the clinical laboratory workflow can be fine tuned with the help of near-at-hand decision-support tools including clinical laboratory support and emerging techniques such as machine learning algorithms and immunophenotyping (6). There is a recent observation from the United Kingdom that laboratory-enhanced surveillance of Gram negative bloodstream infections can predict associated in-hospital mortality (7). However, a teaching hospital’s acronym-laden, jargon-charged specialists may be more than a short ambulance ride away from the pre-hospital and laboratory-free point of sepsis care. In local experience, long distances between major settlements stretch clinical services and their laboratory support beyond reasonable limits so that the patient with suspected sepsis and their pathology specimens have to be transferred out. Patient and specimens may end up travelling in the opposite direction, such is the malalignment of pathology support with clinical care. Highly centralised in-patient services beloved of health administrators include centralised blood culture processing. This guarantees that laboratory results from remote regional patients will not inform treatment decisions in the critical first 48 h of sepsis management. Recent results from the French national blood culture observatory reveal a higher risk of delayed blood culture results over weekends (1). A recent study in the United Kingdom showed that prolonged pre-incubation delays correlated with longer hospital stays (8). Locally, additional delays processing blood cultures from regional centres were exposed by trials of multiplex PCR methods for regional blood culture identification (9). The worst aligned laboratory results come from antimicrobial susceptibility tests, which currently rely on at least two rounds of culture, and often three. Unfortunately, the more resistant the bacterial isolate, the longer a definitive result will take. The time-saving approach taken by laboratory medicine is to use DNA markers of antimicrobial resistance, which deliver a much faster indicator of potential treatment failure, though there is considerable variation on how this approach is implemented in clinical laboratories (10). However, these molecular methods do not predict what antimicrobial agents are suitable for successful treatment. That requires application of agreed interpretive criteria to obtain an antimicrobial susceptibility phenotype (11). No matter how rapid the susceptibility test method, its reliance on prior bacterial growth means a series of preliminary laboratory steps are needed including bacterial identification before definitive prescriber guidance is available.

### Syntax variations

There is an implicit need to understand how to develop actionable information in a timely and orderly manner, and identify where unsupportable informal terminology has crept into common usage. Key decisions in the sepsis patient’s journey need to follow a series of generic steps in the correct order [Are they sick? How severely sick? What is the underlying cause? (12)]. Data scientists and programmers are experts in syntax. Their attention to detailed rendition of code, its order, punctuation and iterations underpin much of the analysis built into algorithms that drive the computer operated systems we use in the clinic, on the ward and in the laboratory. Those algorithms are agnostic of the untranslated differences between clinical sepsis speak and laboratory language. Some early attempts at using machine learning to find a way out of the sepsis bind were a little clunky, and were hampered by under-coding when benchmarked by the current International Classification of Diseases (13). More recent attempts to achieve specific types of clinical decision support, such as prediction of positive blood culture results and immunophenotyping (6, 14), are starting to look useful. A valuable feature of supervised machine learning is its ability to prevent the user from jumping to conclusions before the data has been acquired, curated, classified and visualised. In other words, machine learning leads to actionable data through a reproducible automated process, and can highlight notable outliers. Though this field is full of non-linear complexity, data-driven clinical decisions are within our reach, particularly when multiple sequential data sets are available for analysis (4). Sometimes the data needed for critical decision support in sepsis management is so obvious that it only needs the results from a robust point of care test (15). In our remote regional settings, clinical care of patients with suspected sepsis could be by improved access to a point of care full blood count and C-reactive protein. To press the point on health inequities, we should be starting at the poorly served remote periphery then following the patient’s journey towards the teaching centres; not just working outwards from the expert centre.

### Of quests and errands

The search for an early detection method for sepsis has been something of a heroic quest. For some this has become a fool’s errand. To date, the lack of a single reliable laboratory marker for sepsis has led to multi-biomarker assessment of host response (16). This laboratory-centric approach is unsuitable for the point of care setting, and is thus unlikely to help our need for early prediction of bloodstream infection. We may be close to a screening test for bacterial BSI based on machine learning (14), but at present it lacks the specificity of culture-based techniques. For the meantime, the clinical microbiology laboratory will continue to provide not-quite definitive results for some patients at considerable effort and cost. It would be tragic to exhaust all our efforts on another consensus definition of sepsis (17), yet lose sight of the need for early diagnosis and effective targeted therapy. At this year’s European Congress on Clinical Microbiology and Infectious Diseases, one speaker highlighted the challenge of sepsis’s multilayered complexity and recommended listeners ‘focus on treatable biological traits’ (1). That is not an admission of failure. Rather, it is realistic advice on how best to achieve better clinical outcomes. The emerging common language of infection science integrates clinical infectious diseases, laboratory science, epidemiology, pathophysiology and therapeutic interventions into a practical heuristic (18). Wider acceptance of a pragmatic sepsis heuristic and its corresponding disease phenotype ontology and epistemology would improve the consistency of inter-disciplinary communication on sepsis. The pursuit of clinical service excellence and patient welfare is no less worthwhile than giving a taxonomically correct name to the causal agent of bloodstream infection or recognising an eponymous syndrome. At present, the improvement of sepsis outcomes demands better inter-disciplinary communication, comprehensible language and a helping hand from data science.

## Data availability statement

The original contributions presented in the study are included in the article/supplementary material, further inquiries can be directed to the corresponding author.

## Author contributions

The author confirms being the sole contributor of this work and has approved it for publication.
